# Haematuria Increases Progression of Advanced Proteinuric Kidney Disease

**DOI:** 10.1371/journal.pone.0128575

**Published:** 2015-05-27

**Authors:** Claudia Yuste, Alfonso Rubio-Navarro, Daniel Barraca, Inés Aragoncillo, Almudena Vega, Soraya Abad, Alba Santos, Nicolás Macias, Ignacio Mahillo, Eduardo Gutiérrez, Manuel Praga, Jesús Egido, Juan Manuel López-Gómez, Juan Antonio Moreno

**Affiliations:** 1 Renal Unit. Gregorio Marañón Hospital, Madrid, Spain; 2 Renal, Vascular and Diabetes Research Lab. IIS-Fundación Jiménez Díaz, Autonoma University, Madrid, Spain; 3 Department of Epidemiology. IIS-Fundación Jiménez Díaz, Madrid, Spain; 4 Department of Nephrology. Doce de Octubre Hospital, Madrid, Spain; 5 Centro de Investigación Biomédica en Red de Diabetes y Enfermedades Metabólicas Asociadas (CIBERDEM), Madrid, Spain; 6 Fundación Renal Iñigo Alvarez de Toledo-Instituto Reina Sofía de Investigaciones Nefrológicas (FRIAT-IRSIN), Madrid, Spain; National Cancer Institute, UNITED STATES

## Abstract

**Background:**

Haematuria has been traditionally considered as a benign hallmark of some glomerular diseases; however new studies show that haematuria may decrease renal function.

**Objective:**

To determine the influence of haematuria on the rate of chronic kidney disease (CKD) progression in 71 proteinuric patients with advanced CKD (baseline eGFR <30 mL/min) during 12 months of follow-up.

**Results:**

The mean rate of decline in eGFR was higher in patients with both haematuria and proteinuria (haemoproteinuria, HP, n=31) than in patients with proteinuria alone (P patients, n=40) (-3.8±8.9 vs 0.9±9.5 mL/min/1.73m2/year, p<0.05, respectively). The deleterious effect of haematuria on rate of decline in eGFR was observed in patients <65 years (-6.8±9.9 (HP) vs. 0.1±11.7 (P) mL/min/1.73m2/year, p<0.05), but not in patients >65 years (-1.2±6.8 (HP) vs. 1.5±7.7 (P) mL/min/1.73m2/year). Furthermore, the harmful effect of haematuria on eGFR slope was found patients with proteinuria >0.5 g/24 h (-5.8±6.4 (HP) vs. -1.37± 7.9 (P) mL/min/1.73m2/year, p<0.05), whereas no significant differences were found in patients with proteinuria < 0.5 g/24 h (-0.62±7.4 (HP) vs. 3.4±11.1 (P) mL/min/1.73m2/year). Multivariate analysis reported that presence of haematuria was significantly and independently associated with eGFR deterioration after adjusting for traditional risk factors, including age, serum phosphate, mean proteinuria and mean serum PTH (β=-4.316, p=0.025).

**Conclusions:**

The presence of haematuria is closely associated with a faster decrease in renal function in advanced proteinuric CKD patients, especially in younger CKD patients with high proteinuria levels; therefore this high risk subgroup of patients would benefit of intensive medical surveillance and treatment.

## Introduction

Chronic kidney disease (CKD) is a global public health problem, as result of its growing prevalence and costs, and the higher risk to progress to end-stage renal disease (ESRD), cardiovascular disease and death [1, 2]. The rate of progression of CKD may be affected by several factors [3], including age, gender, proteinuria, hypertension, diabetes mellitus and mineral-bone metabolism biomarkers (fibroblastic growth factor 23, serum phosphate and parathyroid hormone (PTH) levels) [4]. However, these classic risk factors cannot account for some findings. Therefore, it is necessary to identify more relevant risk markers to identify those patients at higher risk to progress faster to ESRD.

Haematuria is defined as the presence of red blood cells (RBCs) in urine. Haematuria is a common finding in various glomerular diseases, such as IgA nephropathy (IgAN), Alport syndrome and thin basement membrane disease [5]; however its presence is usually not mentioned in large epidemiological studies and little is known about its role on CKD progression [6–7]. After decades of considering haematuria as a benign clinical manifestation of glomerular diseases, without real consequences on renal function and long-term prognosis, new evidences pointed its negative implications on the progression of renal disease [8–9]. Thus, persistent asymptomatic isolated microscopic haematuria was significantly associated with increased risk of ESRD in 1 million young Israeli adults after more than 20 years of follow up [8]. In addition, persistent glomerular haematuria in kidney donors was associated with CKD progression at 2.3 years after donation [10]. Negative association between macroscopic haematuria and long-term renal prognosis have been also reported in IgAN patients [9, 11, 12], although there are contradictory results [9,13–15]. On the other hand, it is well known that macroscopic haematuria may leads acute kidney injury (AKI) in IgAN patients and excessively anticoagulated patients (INR>3.0, the so-called warfarin-related nephropathy (WRN)), with an incidence of 30% and 20%, respectively [9,16]. Interestingly, recent evidences show that up to 25% of IgAN patients and 66% WRN patients did not recover baseline renal function after macroscopic haematuria-associated AKI, leading to adverse long-term outcomes [9, 16]. Furthermore, the KDOQI (Kidney Disease Outcomes Quality Initiative) and KDIGO (Kidney Disease: Improving Global Outcome) guidelines recommend to assess every CKD patient with dipstick [17], although they not recommend further monitoring or treatment in patients with glomerulonephritis and isolated microscopic haematuria. However, these guidelines acknowledge that IgAN with haematuria and minimal proteinuria is a progressive disease [18]. In contrast, in ANCA-associated vasculitis, persistent microscopic haematuria did not show a clear repercussion on glomerular filtration rate at 1 year follow up [19]. Therefore, the real impact of haematuria on CKD progression remains unknown.

To date, the relation between the presence of haematuria and decline of renal function has been analyzed in subjects at early CKD stages, excluding systematically to those patients with estimated glomerular filtration rate (eGFR) <45 ml/min [9, 20]. However, advanced CKD are of special interest in clinical practice due to their rising prevalence and higher associated comorbidities. For that reason, in the present article we aimed to analyze the effect of haematuria on the rate of progression of renal function in advanced proteinuric CKD patients over one year of follow up.

## Material and Methods

### Patients

In this retrospective observational longitudinal study, we analyzed data from 300 patients with advanced CKD (stages 4 and 5) followed between 2006 and 2010 in the Renal Unit at Gregorio Marañón Hospital, Madrid, Spain [21]. Patients provided written informed consent. Gregorio Marañón Hospital Ethics Committee approved the study. The investigation conforms to the principles outlined in the Declaration of Helsinki. The exclusion criteria were: (1) proteinuria negative (<0.03 g in 24-hour urinary excretion), (2) haematuria data non recorded, (3) follow up fewer than 6 months and fewer than 4 determinations of eGFR (calculated using Modification of Diet in Renal Disease [MDRD] study 4-item equation) [22], (4) baseline eGFR>30mL/min, (5) patients with malignancies or infections during the follow up, and (6) patients with autosomal dominant polycystic kidney disease, urological malignancies or concurrent urinary infection (in order to avoid the possible inference of non-glomerular haematuria over glomerular haematuria detection). Finally 71 patients were included and prospectively followed up for 11.9 ± 5.4 months. Patients were categorized in 2 groups according to the presence of haematuria. Patients were considered haemoproteinuric (HP) when presented both haematuria and proteinuria, whereas proteinuric patients (P) presented proteinuria alone.

Demographic data and laboratory values were recorded along the study period. In each patient, we recorded the values for serum creatinine, proteinuria, phosphate, calcium, uric acid, PTH, and 24-hour urinary excretion of urea nitrogen in each determination to calculate the mean value for each variable over time. Treatment was also recorded, including antihypertensive drugs, statin therapy, erythropoiesis-stimulating agents, and iron supplements. High blood pressure was defined as an average systolic/diastolic blood pressure of 130/85 mmHg or greater or current use of antihypertensive medication, independently of blood pressure levels. Proteinuria was defined as >0.03 g protein levels in 24-hour urinary excretion determination. CKD progression was assessed as the slope of the regression line of all eGFR measurements (mL/min) over time (years) adjusted for body surface (1.73m^2^) and expressed as mL/min/1.73m^2^/year. Therefore, the faster progression has a more negative slope.

### Laboratory Measurements

Freshly voided samples were collected for urinalysis. Laboratory measurements were made using standardized automated methods, as previously reported [20]. The presence of haematuria was assessed with dipstick, and then confirmed with microscopic examination in each patient and every clinical visit. Patients were considered haematuric if presented at least 3 positive determinations.

### Statistical Analysis

Normally distributed values are expressed as mean±SD; non-normally distributed values are expressed as median±IQR. The differences in qualitative variables were examined using the chi-square test for categorical variables. Continuous variables were compared using the Student’s t-test for independent samples after verifying the normality of distribution using the Kolmogorov-Smirnov test or by analysis of variance (ANOVA) when comparing more groups. A linear model was constructed to identify the relationship between different variables. Multivariate analysis (lineal regression) was performed to determine the associations between the eGFR slope and the predisposing risk factors. On the multivariate analysis we used the serum PTH logarithm in order to correct its asymmetric distribution. All statistical analyses were conducted using SPSS for Windows, version 15 (Chicago,Illinois, USA). Statistical significance was considered at p<0.05.

## Results

Demographic, clinical characteristic and baseline biochemical values according to the presence of HP are summarized on Table 1. The mean annual eGFR slope was -1.12±9.38 mL/min/1.73m^2^/year. None of our patients presented documented macrohaematuria during the follow up. The aetiology of CKD was diabetic nephropathy in 19 patients (26.8%, 10 P vs. 9 HP), followed by nephroangiosclerosis in 15 patients (21%, 11 P vs. 4 HP), glomerular disease in 10 patients (14.1%, 5 P vs. 5 HP), tubulointerstitial nephritis in 10 patients (14.1%, 6 P vs. 4 HP) and unknown in 17 patients (24%, 5 P vs. 12 HP). No significant differences in demographical, CKD aetiology or baseline clinical data were observed between HP and P patients. Eleven haematuric patients (32.4%) were under antiplatelet drug therapy whereas 14 non-haematuric patients (37.8%) received this treatment (p = 0.41). During the follow up, 9 patients reached ESRD in the HP group and 8 in the P group (29% HP vs. 20% P; p = 0.27). Interestingly, HP patients presented higher mean rate of decline in eGFR than patients with proteinuria alone (-3.8±8.7 vs. 0.9±9.5 mL/min/1.73m^2^/year), so they progressed significantly faster to ESRD (p = 0.036) (Table 1).

**Table 1:** Characteristics of patients according to the presence of haematuria.

	HP (n = 31)	P (n = 40)	All (n = 71)	P value
**Clinical characteristics**				
Age (years)	62.9±16.9	66.9±13.0	65.1±14.85	0.27
Gender (Male, %)	20(57.5)	23(64.5)	43(56.3)	0.68
Hypertension (Yes, %)	26 (83.9)	36 (90)	62 (87.3)	0.34
Number of antihypertensive drugs (n)	2.1±1.5	2.7±1.5	2.5±1.5	0.11
RAAS inhibitors (Yes, %)	17(50)	22 (61.1)	39 (55.7)	0.24
Diabetes Mellitus (Yes, %)	12 (38.7)	14 (35)	26 (36.6)	0.47
Body mass index (kg/m^2^)	27.2±4.7	29.6±6.6	28.5±5.9	0.09
Charlson comorbidity index	6.03±2.52	5.18±1.75	5.55±2.15	0.10
Mean number of eGFR determinations per patient (n)	7.7±3.2	7.3±2.5	7.5±2.7	0.50
Follow up (months)	11.56±5.74	12.17±5.1	11.9±5.35	0.79
Age (years)	62.9±16.9	66.9±13.0	65.1±14.85	0.27
**Baseline levels**				
Serum creatinine (mg/dL)	3.32±0.64	3.13±0.66	3.2±0.65	0.23
eGFR (mL/min/1.73m^2^)	19.25±5.47	19.78±4.63	19.6±4.99	0.65
Serum phosphate (mg/dL)	5.1 ±5.9	4.1±0.65	4.5±3.9	0.38
Serum PTH(ng/L)	203[101, 421]	166[124, 283]	185[115, 310]	0.83
Serum calcium (mg/dL)	8.67±0.63	8.4±0.77	8.5±0.7	0.10
Serum haemoglobin (g/dL)	12.4±1.7	11.8±1.4	12.1±1.56	0.11
Proteinuria (g/24 h)	1.11±1.69	0.89±1.38	0.98±1.51	0.55
Urinary excretion of urea nitrogen (g/24h)	16.86±5.82	18.08±5.83	17.5±5.8	0.39
**Mean determination during follow up**				
Proteinuria (g/24 h)	1.06±1.33	0.99±1.27	1.02±1.29	0.81
Serum Phosphate (mg/dL)	4.17±0.77	3.98±0.56	4.07±0.66	0.22
Serum PTH (ng/L)	205[118, 466]	216[173, 322]	208[171, 333]	0.83
Mean Annual eGFR slope (mL/min/1.73m^2^/year)	-3.76±8.68	0.92±9.49	-1.12±9.38	0.03

Results are expressed as mean ± SD, n(%) or median [IQR]. HP, Haemoproteinuria; P, proteinuria alone; eGFR, estimated glomerular filtration rate; PTH, parathyroid hormone; RAAS inhibitors, renin angiotensin aldosterone system inhibitors.

**Fig 1:**
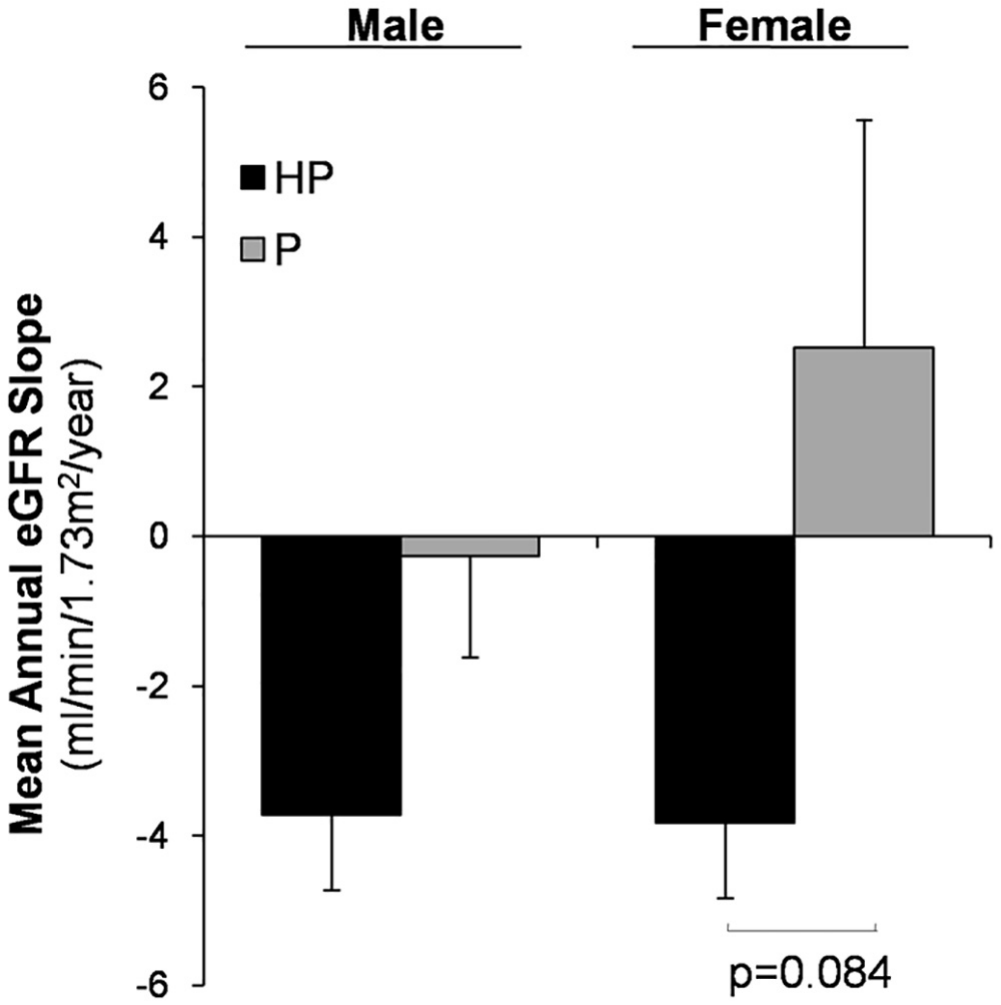
Differences in the mean annual eGFR slope by gender and presence of haematuria. Values are expressed as mean±SD.

We then analyzed whether there was a different effect of haematuria on eGFR decline according to gender. Our results showed that HP patients progressed faster than those with proteinuria alone, independently of gender (Fig 1). Women with HP tended to progress faster than those with proteinuria alone, although no significant differences were found (-3.83±7.3 vs. 2.52±12.5 mL/min/1.73m^2^/year, p = 0.084) (Fig 1).

**Fig 2:**
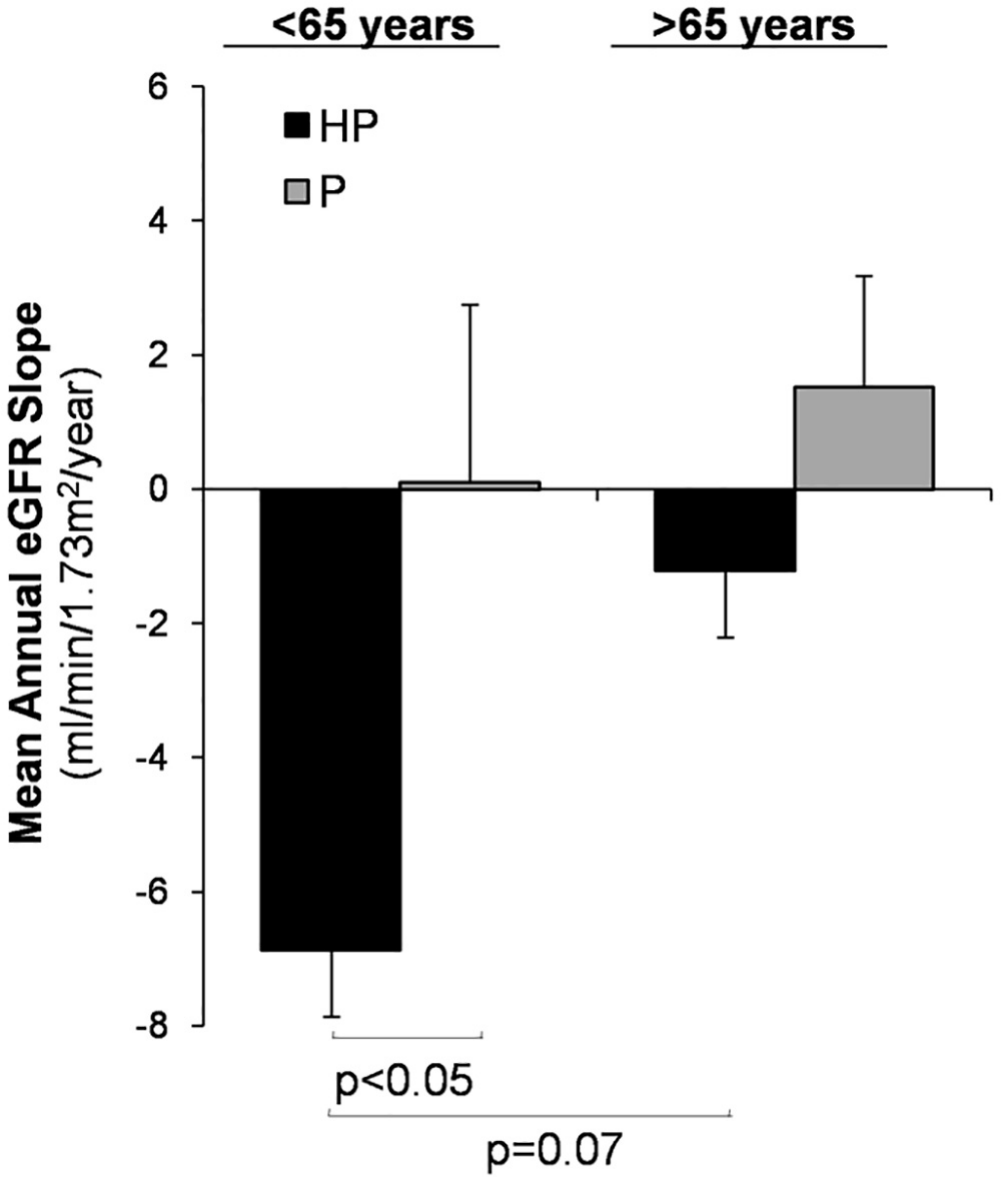
Differences in the mean annual eGFR slope by age and presence of haematuria. NS, non significant. Values are expressed as mean±SD.

We found a significant interaction between the presence of haematuria and age on the progression of CKD. Thus, the effect of haematuria on rate of decline in eGFR was observed in patients <65 years (-6.8±9.9 (HP) vs. 0.1±11.7 (P) mL/min/1.73m^2^/year, p<0.05), but not in patients >65 years (-1.2±6.8 (HP) vs. 1.5±7.7 (P) mL/min/1.73m^2^/year) (Fig 2). Furthermore, eGFR decreased faster in younger HP patients than in older HP patients, although no significant differences were found (−6.8±9.9 vs. -1.2± 6.8 mL/min/1.73m^2^/year respectively, p = 0.07). Patients >65 years with proteinuria alone remained stable or even improved the eGFR slope (Fig 2). We did not observed significant differences in mean proteinuria, serum mean phosphate or serum mean PTH between HP and P groups according to age (Table 2).

**Table 2:** Differences in analytical data by age and presence of haematuria.

Analytical data	<65 years			>65 years		
	HP (n = 14)	P (n = 17)	p value	HP (n = 17)	P (n = 23)	p value
Mean Annual GFR slope (mL/min/1.73m^2^/year)	-6.9±9.9	0.1±11.7	<0.05	-1.2±6.8	1.5±7.68	0.25
Mean proteinuria (g/24 h)	1.37±1.7	1.31±1.27	0.66	0.80±0.9	0.74±1.23	0.87
Mean serum phosphate (mg/dL)	4.29±0.96	4.02±0.50	0.31	4.08±0.58	3.95±0.62	0.50
Mean serum PTH (ng/L)	199 [140, 329]	225 [134, 315]	0.52	207 [118, 474]	214 [179, 333]	0.22

Results are expressed as mean ± SD or median [IQR]

HP, Haemoproteinuria; P, proteinuria alone

**Fig 3:**
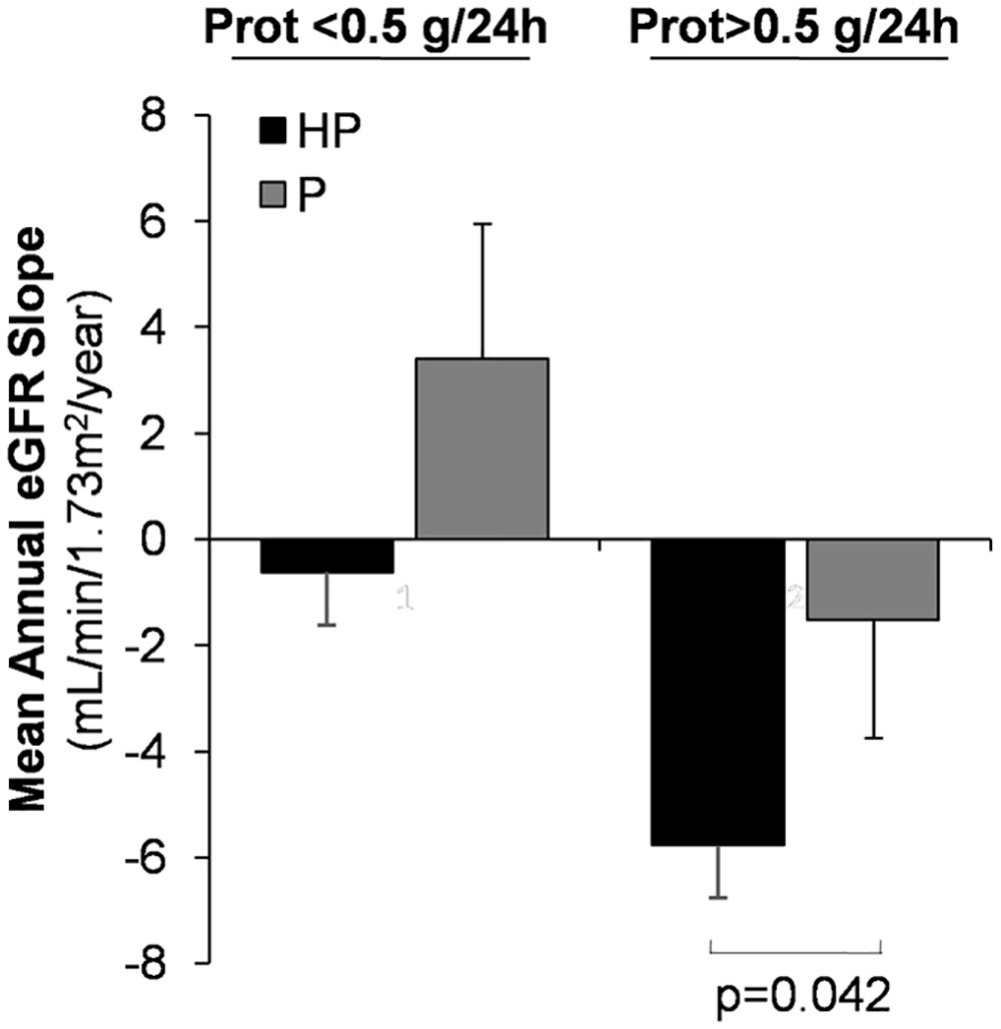
Differences in the mean annual eGFR slope in patients according to proteinuria levels and presence of haematuria. Values are expressed as mean±SD.

To determine whether the effect of haematuria on eGFR slope was associated to proteinuria, our patients were equally distributed into two groups according to the median proteinuria value obtained along the study. Thus, patients were divided into two groups: patients with proteinuria < 0.5 g/24 h or patients with proteinuria > 0.5 g/24 h. The harmful effect of haematuria on eGFR slope was found in patients with proteinuria >0.5 g/24 h (-5.8±6.4 (HP) vs. -1.37± 7.9 (P) mL/min/1.73m^2^/year, p<0.05), whereas no significant difference was found in those patients with proteinuria <0.5 g/24 h (-0.62±7.4 (HP) vs. 3.4±11.1 (P) mL/min/1.73m^2^/year) (Fig 3). No differences were found between haematuric and non haematuric patients in mean proteinuria, serum mean phosphate or serum mean PTH, gender or hypertension according with proteinuria degree (Table 3).

**Table 3:** Differences in analytical data by proteinuria and presence of haematuria.

Analytical data	Proteinuria < 0.5 g/ 24 h			Proteinuria > 0.5g /24 h		
	HP (n = 18)	P (n = 19)	p value	HP (n = 16)	P (n = 17)	p value
Gender (male)	13 (57.9%)	11 (72.2%)	0.49	9(56.3%)	10 (58.8%)	0.58
Hypertension (yes, %)	14 (77.8%)	17 (89.5%)	0.41	14(87.5%)	16(94.1%)	0.6
Mean Annual GFR slope (mL/min/1.73m^2^/year)	-1.5±9.4	3.4±11.1	0.15	-5.7±6.4	-0.62±7.4	0.042
Mean proteinuria (g/24 h)	0.18±0.16	0.12±0.17	0.29	1.85±1.47	1.95±1.13	0.84
Mean serum phosphate (mg/dL)	4.02±0.53	3.9±0.57	0.46	4.05±0.61	4.3±0.9	0.33
Mean serum PTH (ng/L)	234[114, 340]	196 [173, 295]	0.71	232 [178,484]	219 [148, 329]	0.34

Results are expressed as mean ± SD or median [IQR]

HP, Haemoproteinuria; P, proteinuria alone

Univariate analysis reported that progression of eGFR was inversely associated with mean serum phosphate, serum PTH, proteinuria, and haematuria and directly associated with mean serum calcium (Table 4). There were no significant differences for age, gender, hypertension, the number of antihypertensive drugs, and 24-hour urinary excretion of urea nitrogen in the progression of CKD.

**Table 4:** Univariate analysis by Lineal Regression. Factors associated with the rate of eGFR deterioration.

	β	95% CI	p value
Age (years)	0.09	-0.06, 0.24	0.24
Gender (Male)	1.90	-2.655, 6.45	0.41
Hypertension (Yes)	2.07	-4.633, 8.78	0.54
Mean proteinuria (g/24 h)	-2.89	-4.502, -1.29	<0.001
Mean serum phosphate (mg/dL)	-5.24	-8.393, -2.09	0.001
Mean serum PTH (ng/L)	-0.02	-0.03, -0.01	0.010
Mean serum calcium (mg/dL)	4.64	0.37, 8.92	0.033
Urinary excretion of urea nitrogen (g/24h)	0.27	-0.12, 0.66	0.18
Antihypertensives (Yes)	-0.33	-1.81, 1.15	0.66
Haemoproteinuria (Yes)	-4.69	-9.05, -0.32	0.035

PTH: parathyroid hormone.

To clarify whether haematuria was independently associated with eGFR decline in these patients, we performed a multiple regression analysis. Variables that were expected to influence progression of eGFR (mean serum phosphate, serum PTH, proteinuria and mean serum calcium), as well as the presence of haematuria, were included in the multiple regression analysis. In such model, mean proteinuria (β = -2.47 p<0.005), mean serum PTH (β = -4.83, p<0.005) and haematuria (β = -4.316, p<0.05) persisted as independent predictors of eGFR deterioration (Table 5).

**Table 5:** Multivariate analysis by Lineal Regression. Factors associated with the rate of eGFR deterioration.

	β	95% CI	p value
**Mean proteinuria (g/24 h)**	-2.47	-3.96, -0.99	0.001
**Mean serum PTH (ng/L)**	-4.83	-7.82, -1.85	0.002
**Haemoproteinuria (Yes)**	-4.32	-8.09, -0.54	0.026
**Intercept**	29.38	13.29, 45.47	0.001

Adjusted R^2^ = 0.2907. PTH: parathyroid hormone.

## Discussion

In the current study we demonstrated that haematuria is a significant and independent predictor of eGFR deterioration in patients with advanced CKD. Furthermore, we found that the association between haematuria and the rate of decline in eGFR was mainly restricted to younger CKD patients with high proteinuria levels.

Althought hematuria is a common manifestation of glomerular damage, associated with different renal diseases, its relation with CKD progression has traditionally been forgotten in the largest epidemiological studies [6, 7]. This fact may be due to the difficult detection and quantification of glomerular haematuria [23]. Recent reports have highlighted the negative implications of haematuria on renal function deterioration [8, 9]. Thus, it has been reported both acute and long-term worsening of renal function during episodes of macroscopic haematuria in IgAN and WRN patients [9, 16]. In the same line, the largest cohort reported by Vivante et al, showed that persistent asymptomatic isolated microscopic haematuria was significantly associated with increased risk of ESRD after 22 years of follow-up in 1 million young adults [8]. Similarly, persistent glomerular haematuria in kidney donors has been reported as a risk factor for proteinuria and deterioration of renal function at 2.3 years after donation [10]. Goto et al. showed that mild haematuria was associated with the risk of ESRD during 10-year follow-up of IgAN patients and included haematuria in the algorithm to predict renal outcome in these patients [24]. Mild haematuria (1–29 RBCs/high-power field) was the best predictor of renal deterioration in patients with eGFR> 60 mL/min/1.73m^2^, without severe proteinuria (<100 mg/dL), with an OR of 2.3 (95% CI, 1.2–4.3) for renal deterioration at 10-year follow up [25]. Furthermore, disappearance of both haematuria and proteinuria has been proposed as clinical remission markers on IgAN [26] and it is commonly used in clinical trials [20, 27]. In addition, the most influential renal guidelines advised that IgAN with haematuria and minimal proteinuria may be a progressive disease [18]. Nevertheless, there are several contradictory reports, ruling out the repercussion of microhaematuria on the outcome of IgA nephropathy, specially in patients with minimal or no proteinuria [28] or even mild proteinuria [29]. Furthermore, no relation between persistent microscopic haematuria and renal function was reported in patients with ANCA vasculitis [19]. Therefore, the real role of microhaematuria on renal function outcome remains uncertain. In our study, haematuria was significantly associated with a faster progression of renal function decline in advanced CKD patients. Importantly, in our study, the effect of haematuria on eGFR deterioration remained significant after adjusting for traditional risk factors, including proteinuria. This fact may indicate the role of haematuria as a glomerular disease activity marker in patients with chronic renal damage, as reported in advanced CKD patients. To our knowledge, this is the first study analyzing the role of haematuria on the progression of renal disease in patients with advanced CKD.

Haematuria could induce glomerular damage by the cytotoxic, oxidant and inflammatory effects of haemoglobin (Hb) and Hb- related molecules, such, heme and iron [30, 31]. Erythrocyte egression across the glomerular filtration barrier (GFB) induces mechanical stress, leading to a distorted cytoskeleton that is unable to keep Hb lock inside erythrocyte cytoplasm. As consequence, Hb is released from RBC to the urinary space, being then uptaked by tubular epithelial cell where is further transformed into heme and globin. Free heme is extremely toxic, promoting lipid oxidation from intracellular membranes, denaturing proteins, or inducing chemokines release, as monocyte chemoattractant protein [30, 32, 33]. This heme-induced oxidant and inflammatory status could be involved in the faster progression of eGFR decline observed in our study in CKD patients with haematuria. However, new studies are necessary to validate this hypothesis. Importantly, we also found that the harmful effect of haematuria on eGFR slope was maximized in patients with high proteinuria levels. It is probable that proteinuria, by increasing oxidative stress and inflammation, may potentiate the injurious actions promoted by haematuria, thus leading to a more harmful environment that may accelerate progression of renal disease, as we have observed. Therefore, our findings suggest that haematoproteinuric patients would benefit of a more intensive medical surveillance and treatment.

The reduction of eGFR is considered a biological process associated to aging [34]. However, ESRD progression is slow in elderly patients, probably because they present a less aggressive clinical presentation, with low proteinuria, and more easily controlled hypertension [35]. This fact could explain our results, where HP patients > 65 years progressed slowly as compared with the youngest HP patients. Haematuria bouts are much more frequent on early CKD stages of IgAN [14], when glomerulosclerosis is still not present, explaining our findings. A number of reports suggest that haematuria is more intense and recurrent on early CKD stages (1 to 3), pointing glomerular disease activity flares. From these early CKD stages, episodic bouts of haematuria may then progress towards a persistent and low-grade haematuria on advanced CKD, secondary to chronic histological lesions (as fibrosis), maintaining inflammatory stress and promoting CKD progression.

CKD is more frequent in men and male gender has been classically considered as a risk factor for CKD. In agreement with previous reports [8, 36, 37], our data show that the presence of haematuria was more frequent in men. However, we did not find differences on mean annual eGFR slope in HP patients according to gender. Thus, we found that the presence of HP increased the progression of eGFR in both men and women, but significant differences were not reported between genders. Haematuric males presented worse outcome than haematuric females in Alport syndrome, thin glomerular basement nephropathy, C3 glomerulopathy (such CFHR5 nephropathy) [36], and Fabry disease [38], whereas no significant differences were reported in IgAN [37]. Therefore new studies are necessary to address this issue.

It is important to note that the principal limitation of our study is that we could not analyze the effect of the intensity of haematuria on decline of renal function, ie, as a categorical variable quantifying the impact of its intensity. Haematuria was detected by dipstick in the major part of our patients, being unable to quantify the intensity of haematuria. Furthermore, although haematuria was confirmed by microscopic examination in all subjects, quantification of RBC in urine was not reported in a high number of patients. Another limitations of the study are that our results cannot be extrapolated to a more general population, such as patients with less renal damage (CKD stages 1–3), and the small sample size; therefore the results obtained in this study should be confirmed in new studies, with a higher sample size, to corroborate the negative effects of haematuria on progression of renal disease in patients with CKD.

In conclusion, advanced proteinuric CKD patients with haematuria progressed significantly faster to ESRD as compared with patients with proteinuria alone. Younger patients with high proteinuric levels seem to be more sensible to the effect of haematuria. Haematuria, in addition to proteinuria and high serum PTH levels were associated with renal function deterioration. For that reasons it seems mandatory to improve glomerular haematuria detection and the use of quantification techniques to identify patients at higher risk of CKD progression.
